# Characterization of Adipogenic Chemicals in Three Different Cell Culture Systems: Implications for Reproducibility Based on Cell Source and Handling

**DOI:** 10.1038/srep42104

**Published:** 2017-02-08

**Authors:** Christopher D. Kassotis, Lauren Masse, Stephanie Kim, Jennifer J. Schlezinger, Thomas F. Webster, Heather M. Stapleton

**Affiliations:** 1 Nicholas School of the Environment, Duke University, Durham, NC 27708, USA; 2 Department of Environmental Health, Boston University School of Public Health, Boston, MA 02118, USA.

## Abstract

The potential for chemical exposures to exacerbate the development and/or prevalence of metabolic disorders, such as obesity, is currently of great societal concern. Various *in vitro* assays are available to assess adipocyte differentiation, though little work has been done to standardize protocols and compare models effectively. This study compares several adipogenic cell culture systems under a variety of conditions to assess variability in responses. Two sources of 3T3-L1 preadipocytes as well as OP9 preadipocytes were assessed for cell proliferation and triglyceride accumulation following different induction periods and using various tissue culture plates. Both cell line and cell source had a significant impact on potencies and efficacies of adipogenic chemicals. Gene expression analyses suggested that differential expression of nuclear receptors involved in adipogenesis underlie the differences between OP9 and 3T3-L1 cells; however, there were also differences based on 3T3-L1 cell source. Induction period modulated potency and efficacy of response depending on cell line and test chemical, and large variations were observed in triglyceride accumulation and cell proliferation between brands of tissue culture plates. Our results suggest that the selection of a cell system and differentiation protocol significantly impacts the detection of adipogenic chemicals, and therefore, influences reproducibility of these studies.

Both mechanistic laboratory and epidemiological studies implicate exposure to endocrine disrupting chemicals (EDCs) as a factor in many adverse human health trends. EDCs include 1,000 or more synthetic or naturally occurring chemicals or mixtures of chemicals that are able to interfere with hormone action^1^; some of these, termed “metabolic disruptors”, have been shown to directly increase weight gain and/or triglyceride accumulation, and have been reviewed previously^2^. The prevalence of metabolic disorders, such as obesity, is currently of great societal concern^3^,^4^. Obese individuals have an increased risk of type II diabetes, cardiovascular disease, hypertension, and other adverse health effects, and these conditions contribute to more than $215 billion in annual US health care costs^5^.

Due to the extensive costs and time involved in using *in vivo* models, there is a great need to identify and validate appropriate *in vitro* models for screening chemicals that can increase pre-adipocyte proliferation and/or triglyceride accumulation^6^. The 3T3-L1 mouse pre-adipocyte cell line has proven useful as an *in vitro* screen for identifying adipogenic chemicals that can be further assessed *in vivo*. Other model cell lines include the OP9 mouse bone marrow-derived stromal pre-adipocyte cell line^7^,^8^ and various multipotent mesenchymal cells and cell lines^9^,^10^. Following exposure to adipogenic chemicals, these cells differentiate into adipocytes, accumulate triglycerides, and over time develop the characteristics of a mature mammalian white fat cell with a large central lipid droplet and displaced nucleus. Many nuclear receptor systems participate in regulating differentiation of pre-adipocytes and subsequent accumulation of triglycerides, including the peroxisome proliferator-activated receptor-gamma (PPARγ), thyroid receptor-beta (TRβ), glucocorticoid receptor (GR), estrogen receptor (ER), androgen receptor (AR), liver X receptor (LXR), retinoid X receptor (RXR), and others^11^. EDCs that can impact these receptors include a diversity of chemical classes^12^,^13^,^14^, many of which are ubiquitously detected in indoor environments and in human tissues^15^,^16^,^17^,^18^,^19^,^20^,^21^. While many studies have assessed environmental contaminants for receptor activities, far fewer chemicals have been tested for adipogenic capability.

Despite recent interest in evaluating adipogenic activity of chemicals *in vitro*, little work has been done to standardize protocols and comprehensively assess factors that might contribute to disparate degrees of differentiation success between laboratories. Zebisch *et al*. previously described issues with various cell bank stocks of 3T3-L1 cells and reported a decline in degree of differentiation between passages 6 and 10 of ATCC 3T3-L1 cells^22^, in contrast with other researchers that reported robust differentiation through 20–30 passages^7^. This issue may be explained or compounded by many researchers failing to report the source of 3T3-L1 cells utilized^23^,^24^,^25^. Mehra *et al*. further reported that both cell culture vessel size and the proprietary tissue culture coating contributed to the differentiation success of 3T3-L1 cells^26^, though this study only assessed petri dishes and 6-well plates, rarely used today due to inadequate throughput. Lastly, suppliers of these cells have highlighted various factors as important for eliciting maximal differentiation, but these are typically provided without adequate data and rationale^27^,^28^. Comprehensive evaluation of these commonly used cell lines and sources have never been performed to evaluate these variances with the aim of improving reproducibility of adipogenic data between laboratories, particularly in different sources of 3T3-L1 cells and OP9 cells as assessed herein, and using the higher-throughput cell culture dishes utilized by current studies. These assessments are needed to help standardize approaches moving forward and ensure that data generated by multiple laboratories can be compared in a systematic manner, and allow for a greater screening of chemicals.

As such, the goals of this study were to address these disparities and test several adipogenic cell culture systems under a variety of controlled conditions to assess potential differences between cell lines and mechanisms. Specifically, two sources of 3T3-L1 cells were evaluated (American Type Culture Collection, ATCC vs. Zenbio, Inc.) as well as OP9 cells, testing different induction periods and using various tissue culture plate brands. Each cell line was then treated with ligands for nuclear receptors involved in adipogenesis, and gene expression analysis was performed to compare nuclear receptor expression between systems. We hypothesized that these differing cell lines and sources, induction periods, and differentiation supplies would all contribute to variances in the degree of differentiation (triglyceride accumulation and/or cell proliferation) for various test chemicals and possibly lead to mischaracterization of adipogenic compounds.

## Results

### Inconsistencies in adipogenic responses due to varying lengths of exposure

**Figure 1:**
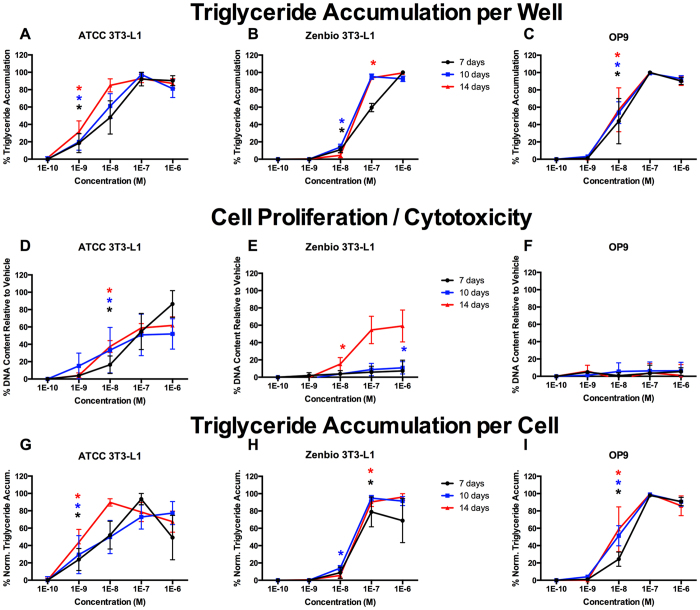
Rosiglitazone Induces Varied Adipogenic Inhibition Based on Induction Time. ATCC 3T3-L1, Zenbio 3T3-L1, and OP9 cells were differentiated as described in Methods and assessed for adipocyte differentiation (Nile Red staining of lipid accumulation) and cell proliferation (Hoechst staining) at various times after initiation of differentiation. Percent raw triglyceride accumulation per well relative to maximal response for rosiglitazone (RSG) at 7 days (**A**), 10 days (**B**), and 14 days (**C**). Increase (cell proliferation) or decrease (potential cytotoxicity) in DNA content relative to vehicle control for RSG at 7 days (**D**), 10 days (**E**), and 14 days (**F**). Percent normalized triglyceride accumulation per cell (normalized to DNA content) for RSG at 7 days (**G**), 10 days (**H**), and 14 days (**I**). Data presented as mean ± SE from three independent experiments. *Indicates lowest concentration with significant increase in triglyceride over vehicle control, p < 0.05, as per linear mixed model in SAS 9.4.

Well-described control chemicals (rosiglitazone (RSG), a PPARγ agonist; tributyltin chloride (TBT), a PPARγ/RXR agonist; T0070907 and GW9662, PPARγ antagonists) were first assessed in each cell line to determine the effect of different induction periods on adipogenic activity. In this set of experiments we evaluated the effects of induction with known controls at 7, 10, and 14 days in each cell line to determine the optimal incubation periods for evaluating adipocyte differentiation (triglyceride accumulation) and cell proliferation (based on DNA content). RSG exhibited more potent responses (lower EC20/50 values; concentrations that exhibit 20/50% of maximal activity) with increased induction time in ATCC 3T3-L1 cells (Fig. 1A,G), 10 and 14 days were equivalent but more potent than 7 days in Zenbio 3T3-L1 cells (Fig. 1B,H), and no differences across days were observed in OP9 cells (Fig. 1C,I). Each induction time stimulated equivalent cell proliferation in ATCC 3T3-L1 cells (Fig. 1D), while Zenbio 3T3-L1 cells exhibited a significantly greater proliferative response at 14 days (Fig. 1E). Induction time had no effect on cell proliferation in OP9 cells (Fig. 1F, Supplemental Figures 1–3).

**Figure 2:**
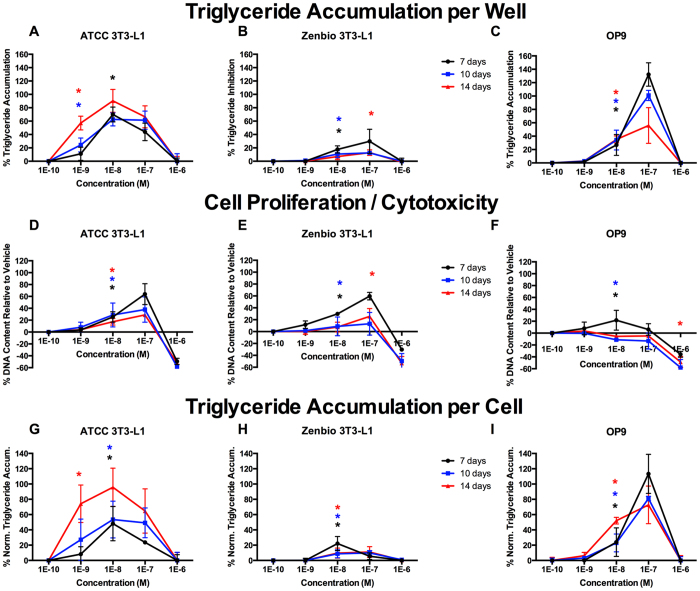
Tributyltin Chloride Induces Varied Adipogenic Activities Based on Induction Time. ATCC 3T3-L1, Zenbio 3T3-L1, and OP9 cells were differentiated as described in Methods and assessed for adipocyte differentiation (Nile Red staining of lipid accumulation) and cell proliferation (Hoechst staining) at various times after initiation of differentiation. Percent raw triglyceride accumulation per well relative to maximal rosiglitazone response for tributyltin chloride (TBT) at 7 days (**A**), 10 days (**B**), and 14 days (**C**). Increase (cell proliferation) or decrease (potential cytotoxicity) in DNA content relative to vehicle control for TBT at 7 days (**D**), 10 days (**E**), and 14 days (**F**). Percent normalized triglyceride accumulation per cell (normalized to DNA content) for TBT at 7 days (**G**), 10 days (**H**), and 14 days (**I**). Triglyceride accumulation responses are provided as relative activity to maximal rosiglitazone. Data presented as mean ± SE from three independent experiments. *Indicates lowest concentration with significant increase in triglyceride over vehicle control, p < 0.05, as per linear mixed model in SAS 9.4.

Triglyceride accumulation stimulated by TBT exposure exhibited markedly different responses between cell lines based on induction time (Fig. 2). Specifically, more triglyceride accumulation was observed with longer induction times in ATCC 3T3-L1 cells (Fig. 2A,G), but was greater with shorter induction times in Zenbio 3T3-LI and OP9 cells (Fig. 2B,C,H,I). The greatest proliferative response was observed at 7 days in all cell lines (Fig. 2D–F). While TBT induced a proliferative response at 10 nM in OP9 cells at 7 days, it did not increase proliferation at 10 and 14 days (Figs 2F, S1–S3).

**Figure 3:**
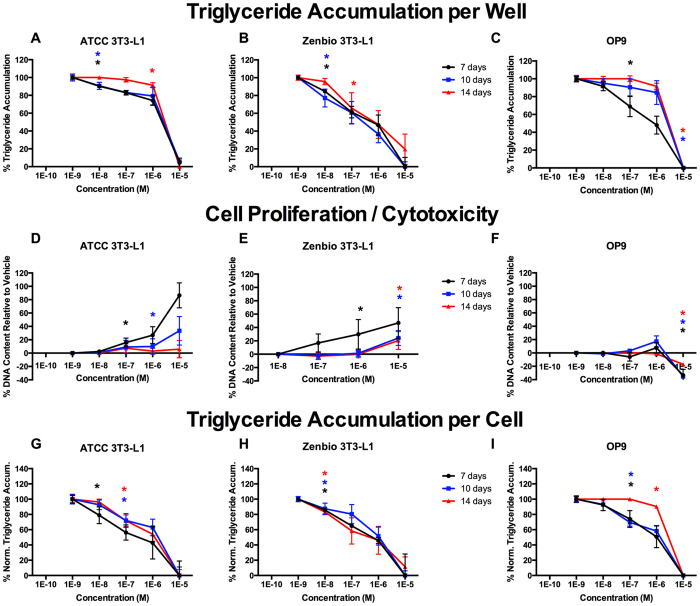
T0070907 Induces Varied Adipogenic Inhibition Based on Induction Time. ATCC 3T3-L1, Zenbio 3T3-L1, and OP9 cells were differentiated as described in Methods and assessed for adipocyte differentiation (Nile Red staining of lipid accumulation) and cell proliferation (Hoechst staining) at various times after initiation of differentiation. Percent raw triglyceride inhibition of half maximal rosiglitazone per well for T0070907 at 7 days (**A**), 10 days (**B**), and 14 days (**C**). Increase (cell proliferation) or decrease (potential cytotoxicity) in DNA content relative to vehicle control for T0070907 at 7 days (**D**), 10 days (**E**), and 14 days (**F**). Percent normalized triglyceride accumulation per cell (normalized to DNA content) for T0070907 at 7 days (**G**), 10 days (**H**), and 14 days (**I**). Data presented as mean ± SE from three independent experiments. *Indicates lowest concentration with significant increase in triglyceride over vehicle control, p < 0.05, as per linear mixed model in SAS 9.4.

Inhibition of triglyceride accumulation by T0070907 exposure also varied considerably between cell lines (Fig. 3). Generally, less potent responses were observed with longer induction times; ATCC 3T3-L1 cells exhibited no difference at 7 and 10 days (Fig. 3A,G), but T0070907 was significantly less potent at 14 days. No differences were observed at any induction time in Zenbio 3T3-L1 cells (Fig. 3B,H). Significantly greater inhibition was observed at 7 days relative to 10 or 14 in OP9 cells (Fig. 3C,I). Markedly different responses were observed in cell proliferation. Less proliferation was observed with longer induction times in ATCC and Zenbio 3T3-L1 cells (Fig. 3D,E). There was no increase in proliferation in OP9 cells early in differentiation, although there was potential cell-specific cytotoxicity at the highest concentration (Fig. 3F); this may be due to an induction of apoptosis via oxidative stress in immature adipocytes, which has been reported previously for T0070907 in 3T3-L1 cells^29^.

**Figure 4:**
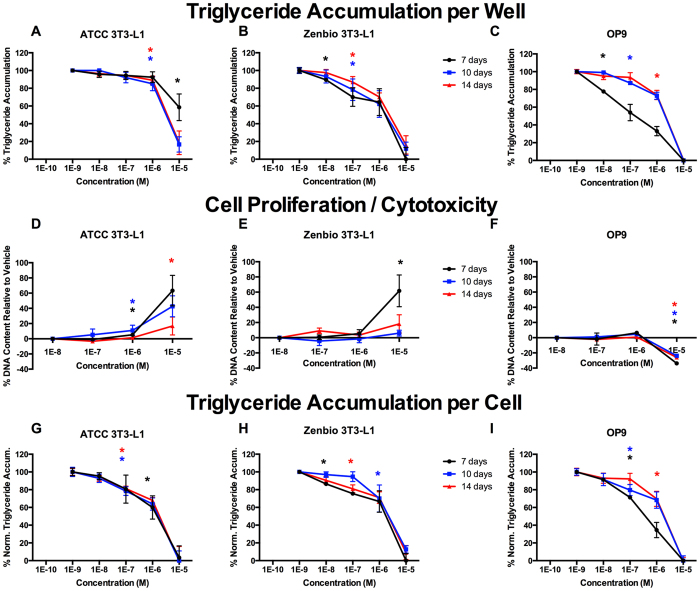
GW9662 Induces Varied Adipogenic Inhibition Based on Induction Time. ATCC 3T3-L1, Zenbio 3T3-L1, and OP9 cells were differentiated as described in Methods and assessed for adipocyte differentiation (Nile Red staining of lipid accumulation) and cell proliferation (Hoechst staining) at various times after initiation of differentiation. Percent raw triglyceride inhibition of half maximal rosiglitazone per well for GW9662 at 7 days (**A**), 10 days (**B**), and 14 days (**C**). Increase (cell proliferation) or decrease (potential cytotoxicity) in DNA content relative to vehicle control for GW9662 at 7 days (**D**), 10 days (**E**), and 14 days (**F**). Percent normalized triglyceride accumulation per cell (normalized to DNA content) for GW9662 at 7 days (**G**), 10 days (**H**), and 14 days (**I**). Data presented as mean ± SE from three independent experiments. *Indicates lowest concentration with significant increase in triglyceride over vehicle control, p < 0.05, as per linear mixed model in SAS 9.4.

Inhibition of triglyceride accumulation by GW9662 varied considerably between cell lines (Fig. 4). Longer induction times were necessary for complete inhibition in ATCC 3T3-L1 cells (Fig. 4A), though no differences across induction times were apparent in Zenbio 3T3-L1 cells (Fig. 4B). Greater inhibitory potency was observed at 7 days relative to 10 and 14 days in OP9 cells (Fig. 4C). Increased cell proliferation was observed at shorter induction times in ATCC and Zenbio 3T3-L1 cells (Fig. 4D,E). Similar to T0070907, apparent cytotoxicity was observed in OP9 cells (Fig. 4F). Fewer differences were apparent in 3T3-L1 responses relative to induction times once triglyceride accumulation was normalized to DNA content, though these effects persisted in OP9 cells (Fig. 4G–I).

### Inconsistencies in Responses Based on Tissue Culture Plate Source

**Figure 5:**
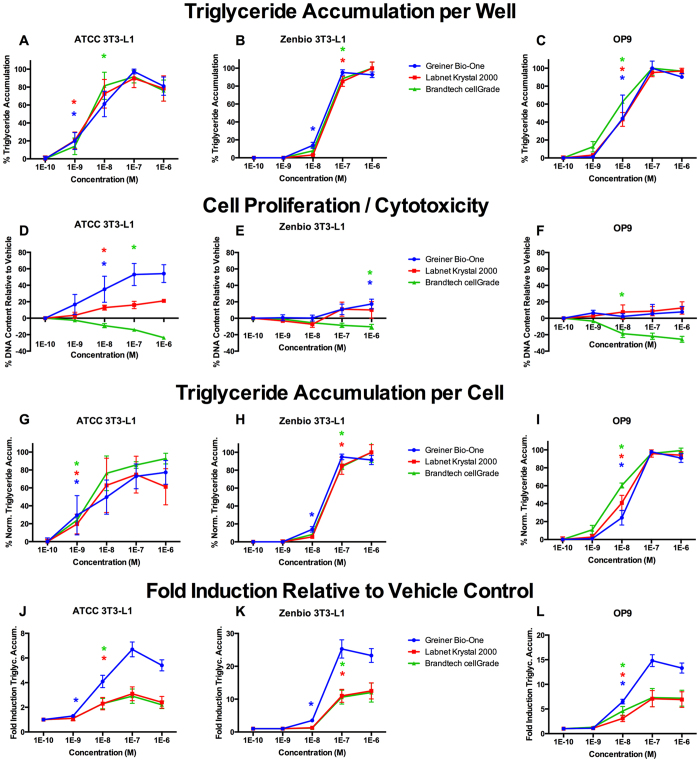
Rosiglitazone Induces Varied Adipogenic Activities Based on Cell Culture Plastic. ATCC 3T3-L1, Zenbio 3T3-L1, and OP9 cells were differentiated as described in Methods and assessed for adipocyte differentiation (Nile Red staining of lipid accumulation) and cell proliferation (Hoechst staining) using various tissue culture plates. Percent raw triglyceride accumulation per well relative to maximal rosiglitazone response for rosiglitazone cultured in Greiner Bio-One CELLSTAR™, Brandtech cellGrade™, and Labnet International Krystal™ 2000 tissue culture plates for ATCC 3T3-L1 cells (**A**), Zenbio 3T3-L1 cells (**B**), and OP9 cells (**C**). Increase (cell proliferation) or decrease (potential cytotoxicity) in DNA content relative to vehicle control for rosiglitazone cultured in the tissue culture plates described above for ATCC 3T3-L1 cells (**D**), Zenbio 3T3-L1 cells (**E**), and OP9 cells (**F**). Percent normalized triglyceride accumulation per cell (normalized to DNA content) for rosiglitazone cultured in the tissue culture plates described above for ATCC 3T3-L1 cells (**G**), Zenbio 3T3-L1 cells (**H**), and OP9 cells (**I**). Fold induction of triglyceride accumulative response over vehicle control for rosiglitazone cultured in the cell culture plastics described above for ATCC 3T3-L1 cells (**J**), Zenbio 3T3-L1 cells (**K**), and OP9 cells (**L**). Data presented as mean ± SE from three independent experiments. *Indicates lowest concentration with significant increase in triglyceride over vehicle control, p < 0.05, as per linear mixed model in SAS 9.4.

RSG responses were further assessed in three brands of black, clear-bottom 96-well tissue culture plates to assess their potential influence on differentiation and cell proliferation. In these experiments, cells were exposed for 7 days (OP9 cells) or 10 days (3T3-L1 cells), induction periods found to be optimal in the experiments above, in three brands of 96-well polystyrene tissue culture plates: Greiner Bio-One CELLSTAR™, Brandtech cellGrade™, and Labnet Krystal™ 2000. RSG-exposed ATCC and Zenbio 3T3-L1 cells in Greiner plates exhibited significant triglyceride accumulation at 1 nM, with lower potencies observed in Brandtech and Labnet plates (Fig. 5A,B), though this was not apparent in OP9 cells (Fig. 5C). ATCC 3T3-L1 cells in Greiner plates exhibited significantly greater proliferation in response to RSG than those in Krystal plates. RSG caused a decrease in ATCC 3T3-L1 DNA content in Brandtech plates (Fig. 5D). The same trends were apparent but less pronounced in Zenbio 3T3-L1 cells: RSG induced cell proliferation at 1 μM only in the Greiner plates, no proliferation was observed in Krystal plates, and apparent plate-specific cytotoxicity was observed in Brandtech plates (Fig. 5E). In OP9 cells, no RSG-induced proliferation was observed in Greiner or Krystal plates, but RSG similarly exhibited cytotoxicity in the Brandtech plates (Fig. 5F). Differences in cell proliferation contributed to improved sensitivity for triglyceride accumulation (increased potency) in ATCC 3T3-L1 cells (Fig. 5G–I), potentially due to the lower relative fold inductions in ATCC cells and compounded with the decreased responses observed in Brandtech and Labnet plates (Fig. 5J–L).

### Inconsistencies in adipogenic responses between cell lines and cell sources

Following optimization of the differentiation protocol (selecting variables that elicited the most potent and efficacious responses; 7 day induction for OP9 and 10 for 3T3-L1, Greiner tissue culture plates), cell lines and sources were compared to evaluate differences in adipogenic responses. The first set of these experiments assessed the triglyceride accumulation and cell proliferative responses to RSG, TBT, T0070907, and GW9662. RSG exhibited consistent responses across cell lines, with slightly greater potency (significant triglyceride accumulation at lower concentrations) in the ATCC 3T3-L1 cells (Table 1, Supplemental Figure 4). However, standard error measurements were greater in ATCC 3T3-L1 cells compared to Zenbio 3T3-L1 or OP9 cells, possibly due to >3-fold greater fold induction relative to vehicle in these lines (Fig. 5G–I). RSG induced large increases in cell proliferation in ATCC 3T3-L1 cells at 10, 100, and 1,000 nM, though this was only apparent at 1,000 nM in Zenbio 3T3-L1 cells, and no increase in proliferation was observed in OP9 cells (Table 1, Figures S1–S4).

TBT exhibited markedly different responses between cell lines; between 10–100 nM, TBT induced approximately 75% of intra-assay maximal RSG-induced triglyceride accumulation in ATCC 3T3-L1 cells, only ~15% in Zenbio 3T3-L1 cells, and 130% in OP9 cells (Table 1, Figure S4). Significant cell proliferation at 10 and 100 nM and cytotoxicity at 1 μM were observed in all three cell lines.

T0070907 and GW9662 acted similarly between cell lines; near or complete inhibition of triglyceride accumulation was observed at 10 μM (Table 1, Supplemental Figure 5), though potencies varied. T0070907 exhibited a 2-fold lower IC_50_ in Zenbio 3T3-L1 and OP9 cells than ATCC 3T3-L1 cells, with a ≤ 10-fold lower IC_20_ (Table 1). In contrast, GW9662 exhibited a 30-fold more potent IC_20_ in OP9 than in ATCC or Zenbio 3T3-L1 cells. Each antagonist elicited cell proliferation in the ATCC 3T3-L1 cells at 1 and 10 μM, only T0070907 induced proliferation in Zenbio 3T3-L1 cells at 10 μM, and both compounds exhibited either cytotoxicity or inhibited cell proliferation in OP9 cells at 10 μM (Table 1, Figure S5). Both antagonists also seemed to induce a concentration dependent cytotoxicity, previously reported for T0070907, which not only inhibits adipogenesis through a PPARγ-dependent mechanism but also induces apoptosis of immature adipocytes via oxidative stress^29^.

**Figure 6:**
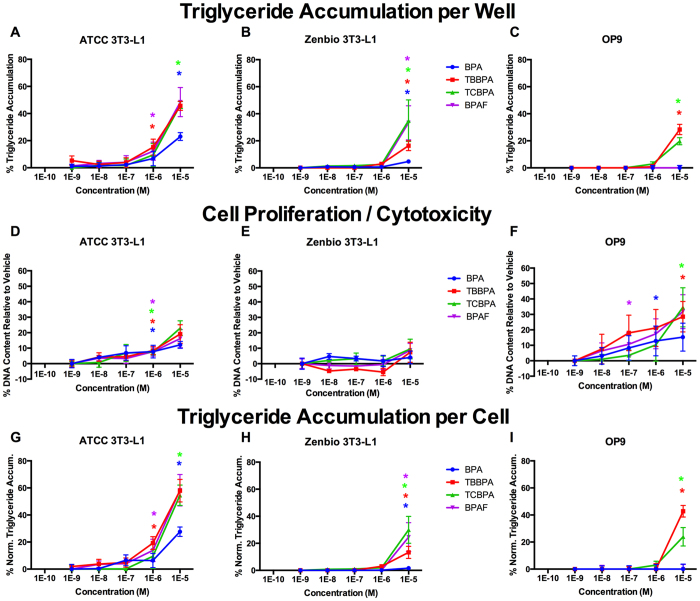
Bisphenol A and Analogs Induce Varied Adipogenic Activities Between Cell Lines. ATCC 3T3-L1, Zenbio 3T3-L1, and OP9 cells were differentiated as described in Methods and assessed for adipocyte differentiation (Nile Red staining of lipid accumulation) and cell proliferation (Hoechst staining) following seven days (OP9) or ten days (3T3-L1) of treatment with four bisphenol A analogs. Percent raw triglyceride accumulation per well relative to maximal rosiglitazone response for bisphenol A (BPA), tetrabrominated bisphenol A (TBBPA), tetrachlorinated bisphenol A (TCBPA), and hexafluorinated bisphenol A (BPAF) in ATCC 3T3-L1 cells (**A**), Zenbio 3T3-L1 cells (**B**), and OP9 cells (**C**). Increase (cell proliferation) or decrease (potential cytotoxicity) in DNA content relative to vehicle control for test chemicals in ATCC 3T3-L1 cells (**D**), Zenbio 3T3-L1 cells (**E**), and OP9 cells (**F**). Percent normalized triglyceride accumulation per cell (normalized to DNA content) for test chemicals in ATCC 3T3-L1 cells (**G**), Zenbio 3T3-L1 cells (**H**), and OP9 cells (**I**). Triglyceride accumulation responses are provided as relative activity to maximal rosiglitazone. Data presented as mean ± SE from three independent experiments. *Indicates lowest concentration with significant increase in triglyceride over vehicle control, p < 0.05, as per linear mixed model in SAS 9.4.

In the next set of experiments, bisphenol A (BPA) and three structural analogs (tetrabrominated BPA, TBBPA; tetrachlorinated BPA, TCBPA; and hexafluorinated BPA, BPAF) were assessed in each cell line, under the optimized conditions discussed above. BPA was the least efficacious compound in each, exhibiting 23% triglyceride accumulation (relative to the maximal rosiglitazone response) in ATCC 3T3-L1 cells, 3% in Zenbio 3T3-L1 cells, and none in OP9 cells (Fig. 6A–C,G–I, Table 1). The halogenated bisphenols, TCBPA, TBBPA, and BPAF exhibited equivalent potencies and efficacies in ATCC 3T3-L1 cells of 45–50% triglyceride accumulation (Fig. 6A,G, Figures S1–S3). TCBPA and BPAF induced ~35% triglyceride accumulation in Zenbio 3T3-L1 cells, although TBBPA induced only 16% (Fig. 6B,H). Surprisingly, BPAF exhibited no activity in OP9 cells, although TBBPA and TCBPA exhibited 28% and 20%, respectively (Fig. 6C,I). All four induced significant cell proliferation in ATCC 3T3-L1 cells, though BPA exhibited the least (Fig. 6D). In contrast, none induced significant proliferation in Zenbio cells (Fig. 6E). Interestingly, while BPA and BPAF did not induce triglyceride accumulation in OP9 cells, all four phenols increased cell proliferation (Fig. 6F).

### Inconsistencies in various receptor-driven adipogenic responses between cell lines

Following these disparate results between cell lines and sources, cells were further assessed by exposing the cells to specific nuclear receptor ligands to determine which receptors were likely contributing to the disparate responses observed. In these experiments, RSG was used to assess PPARγ agonism, GW3965 for LXR agonism, dexamethasone (DEX) for GR agonism, 17β-estradiol (E2) for ER agonism, LG100268 for RXR agonism, 1–850 for TR antagonism, triiodothyronine (T3) for TR agonism, and flutamide for AR antagonism. These ligands/receptor pathways were selected because they were previously reported to play a role in adipocyte differentiation^11^,^30^.

**Figure 7:**
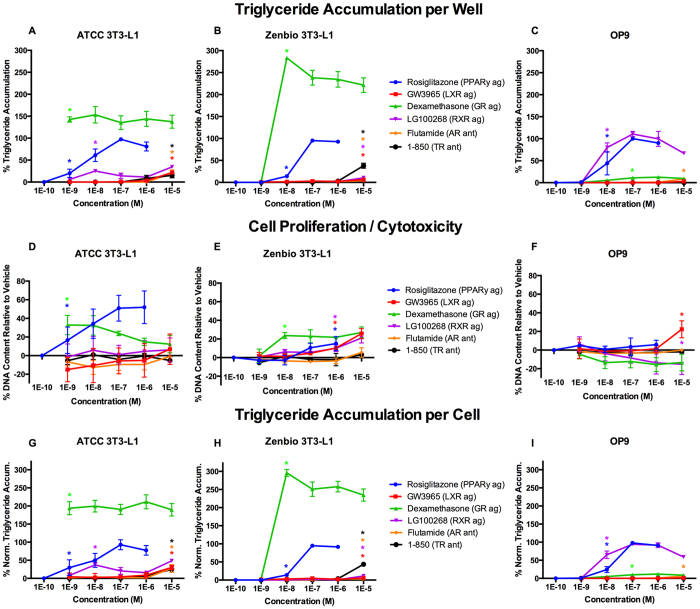
Mechanistic Receptor Controls Induce Varied Adipogenic Activities Between Cell Lines. ATCC 3T3-L1, Zenbio 3T3-L1, and OP9 cells were differentiated as described in Methods and assessed for adipocyte differentiation (Nile Red staining of lipid accumulation) and cell proliferation (Hoechst staining) following seven days (OP9) or ten days (3T3-L1) of treatment with mechanistic receptor control ligands. Percent raw triglyceride accumulation per well relative to maximal rosiglitazone response for rosiglitazone (RSG, PPARγ agonist), GW3965 (liver X receptor, LXR, agonist), dexamethasone (glucocorticoid receptor, GR, agonist), 1–850 (thyroid receptor, TR, antagonist), flutamide (androgen receptor, AR, antagonist), and LG100268 (retinoid X receptor, RXR, agonist) in ATCC 3T3-L1 cells (**A**), Zenbio 3T3-L1 cells (**B**), and OP9 cells (**C**). Increase (cell proliferation) or decrease (potential cytotoxicity) in DNA content relative to vehicle control for test chemicals in ATCC 3T3-L1 cells (**D**), Zenbio 3T3-L1 cells (**E**), and OP9 cells (**F**). Percent normalized triglyceride accumulation per cell (normalized to DNA content) for test chemicals in ATCC 3T3-L1 cells (**G**), Zenbio 3T3-L1 cells (**H**), and OP9 cells (**I**). Responses are provided as relative activity to maximal rosiglitazone. Data presented as mean ± SE from three independent experiments. *Indicates lowest concentration with significant increase in triglyceride over vehicle control, p < 0.05, as per linear mixed model in SAS 9.4.

RSG elicited relatively consistent responses across cell lines as described above. GW3965 induced 22% relative triglyceride accumulation in ATCC 3T3-L1 cells, 8% in Zenbio 3T3-L1 cells, and none in OP9 cells (Fig. 7A–C, G–I, Table S1). DEX induced supramaximal triglyceride accumulation (150% relative to RSG max) at 1 nM in ATCC 3T3-L1 cells, 0% at 1 nM but supramaximal accumulation (300%) at 10 nM in Zenbio 3T3-L1 cells, and 20% at 1 μM in OP9 cells (Fig. 7A–C, G–I, Figures S1–S3). LG100268 induced 35% triglyceride accumulation in ATCC 3T3-L1 cells, 10% in Zenbio cells, and 110% in OP9 cells (Fig. 7A–C, G–I, Figures S1–S3). 1–850 exhibited approximately 15% relative triglyceride accumulation in ATCC 3T3-L1 cells, 50% in Zenbio cells, and none in OP9 cells (Fig. 7A–C, G–I, Table S1). Flutamide exhibited 20% relative triglyceride accumulation in ATCC 3T3-L1 cells, 5% in Zenbio 3T3-L1 cells, and 9% in OP9 cells (Fig. 7A–C, G–I). Estradiol and T3 exhibited minimal or no triglyceride accumulation (Table S1). GW3965 induced cell proliferation in Zenbio 3T3-L1 cells and OP9 cells at 1 and 10 μM, respectively, but none in ATCC 3T3-L1 cells (Fig. 7D–F). DEX induced cell proliferation at equivalent concentrations to triglyceride accumulation; maximal proliferation was induced at 1 nM in ATCC 3T3-L1 cells, 10 nM in Zenbio 3T3-L1 cells, and none in OP9 cells (Fig. 7D–F). LG100268 induced cell proliferation at 1 10 μM in Zenbio 3T3-L1 cells, and no proliferation was observed in ATCC 3T3-L1 or OP9 cells (Fig. 7D–F). 1–850, flutamide (Fig. 7D–F), estradiol, and T3 (Table S1) induced no proliferative response in any cell line.

### Inconsistencies in nuclear receptor mRNA expression between cell lines

**Figure 8:**
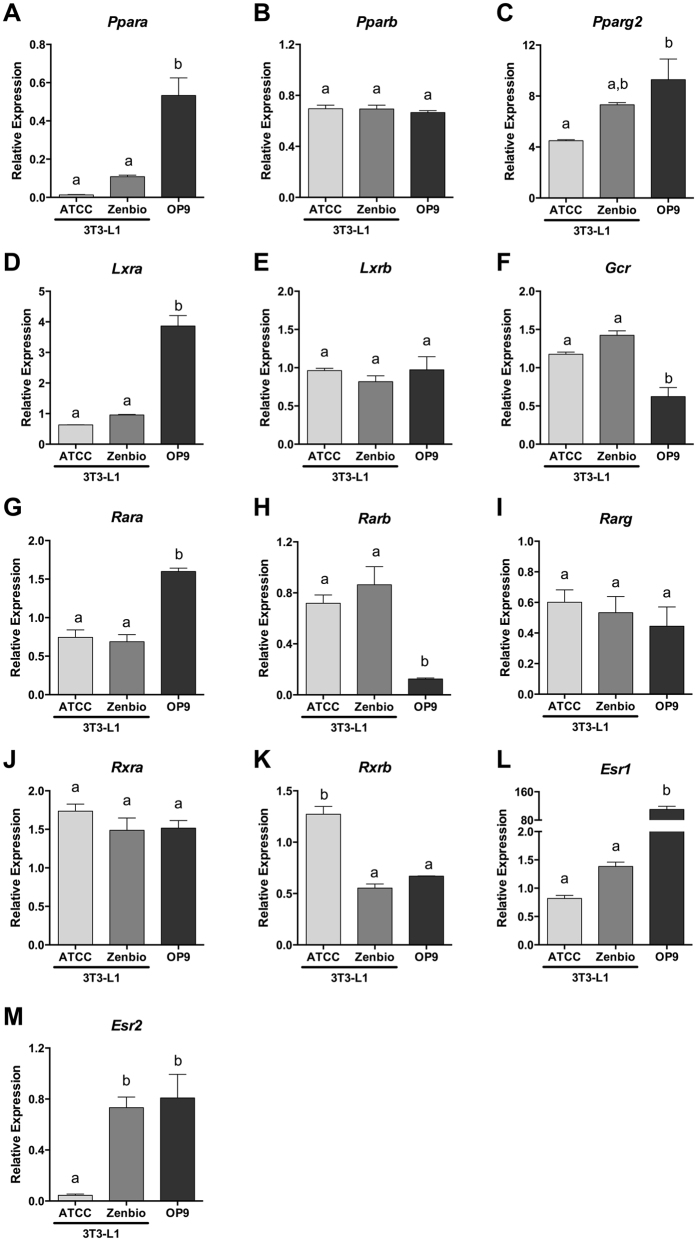
Nuclear Receptor Gene Expression Varies Between Pre-differentiated Cell Lines. ATCC 3T3-L1, Zenbio 3T3-L1, and OP9 cells were cultured as described in Methods. Prior to induction of differentiation, RNA was extracted, cDNA prepared, and qPCR performed on a set of nuclear receptor genes involved in adipogenesis: peroxisome proliferator activated receptor α (PPARα, (**A**), PPARβ (**B**), PPARγ (**C**), liver X receptor α (LXRα; (**D**), LXRβ (**E**), glucocorticoid receptor (**F**), retinoic acid receptor α (RARα; (**G**), RARβ (**H**), RARγ (**I**), retinoid X receptor α (RXRα; (**J**), RXRβ (**K**), estrogen receptor α (ERα, (**L**), ERβ (**M**). Relative expression of each gene provided using 3T3-Swiss Albino cells from ATCC as the reference in three replicate assays. Different letters denote statistically different groups as per one-way ANOVA in GraphPad Prism.

To further assess mechanistic differences in adipogenic signaling pathways, mRNA expression of the nuclear receptors described above were quantified in each of the three un-differentiated cell lines and normalized to expression in 3T3-Swiss Albino cells, the precursor cells for the 3T3-L1 cell line (Fig. 8). Undifferentiated cells were selected to determine nuclear receptors present and available for activation/inhibition by ligands that may drive disparate differentiation responses. PPARα expression was 5–10x higher in OP9 relative to 3T3-L1 cells (Fig. 8A), PPARβ was not different between the cell lines (Fig. 8B), and PPARγ was twice as highly expressed in OP9 cells than ATCC 3T3-L1 (Fig. 8C). Given the greater response of 3T3-L1 cells to GW3965 (LXR agonist), the 4–5-fold greater expression of LXRα in OP9 cells was unexpected, while 3T3-L1 lines were not different (Fig. 8D). LXRβ expression was equivalent between cell lines (Fig. 8E). As expected, given the discordant DEX results, OP9 exhibited significantly lower expression of the glucocorticoid receptor than 3T3-L1 cells (Fig. 8F). RARα was more highly expressed in OP9 cells than 3T3-L1 lines (Fig. 8G), RARβ was more highly expressed in both 3T3-L1 lines than in OP9 cells (Fig. 8H), and RARγ was not different between cell lines (Fig. 8I). RXRγ was expressed in OP9 cells but was not expressed in 3T3-Swiss Albino cells or either 3T3-L1 line used herein and was thus not quantified (data not shown), perhaps explaining the greatly enhanced LG100268 (RXR agonist) activity in OP9 cells. RXRα expression was not different between cell lines (Fig. 8J), and RXRβ was significantly greater in ATCC 3T3-L1 cells relative to both Zenbio 3T3-L1 and OP9 cells (Fig. 8K). ERα expression was 60–100-fold more highly expressed in OP9 cells relative to 3T3-L1 cells (Fig. 8L), while ERβ exhibited significantly greater expression in Zenbio 3T3-L1 and OP9 cells relative to ATCC 3T3-L1 cells (Fig. 8M).

## Discussion

While anecdotal reports exist on variability in the degree of differentiation success based on cell model, time exposed, and cell culture supplies utilized, this is the first study to comprehensively assess these disparities with controlled testing of several adipogenic cell culture systems under a variety of conditions and attempt to determine causative mechanisms. Herein, we have demonstrated that both cell line (3T3-L1 vs. OP9) and cell source (ATCC vs. Zenbio 3T3-L1) have a significant impact on the responses to various chemicals and that differing protocols and supplies utilized may contribute to a lack of reproducibility and bias in measuring adipogenic potency and efficacy of chemicals between laboratories.

Importantly, mechanistic ligand and pre-differentiation gene expression testing demonstrated clear differences between adipogenic receptor pathways in these cell lines. LXR, RXR, GR, and TR ligands resulted in disparate responses between cell lines (Table S1). The LXR agonist, GW3965, exhibited significant but low efficacy in producing triglyceride accumulation in the 3T3-L1 cells, demonstrating that LXR promotes lipogenesis in these cells, but not in OP9, despite greater LXRα expression; this may be due to approximately 10-fold lower binding affinity for LXRα than LXRβ^31^. Likewise, DEX exhibited supramaximal activities in the 3T3-L1 lines but was only minimally active in OP9 cells, consistent with significantly lower GR expression. Interestingly, ATCC 3T3-L1 cells appeared to be more sensitive to DEX treatment, with maximal activity at 1 nM compared to 10 nM in Zenbio 3T3-L1 cells, though Zenbio cells exhibited 2-fold greater relative triglyceride accumulation. The TR antagonist, 1–850, exhibited ~50% relative activity in Zenbio cells, 15% in the ATCC cells, and none in the OP9, clearly demonstrating a divergent response in TR signaling between these cell lines. Lastly, RXR response was very different between these cells, with supramaximal activity (111%) with LG100268 in OP9 cells, 35% in ATCC cells, and 11% in Zenbio cells. Interestingly, while RXRα and β were detected in all cell lines, RXRγ only was detectable in OP9 cells. While maximal PPARγ responses were used to normalize adipogenic results, gene expression testing revealed that PPARγ was more highly expressed in OP9 than ATCC 3T3-L1 cells; this may partly explain the lower relative fold induction in these cells, though Zenbio 3T3-L1 cells had the greatest relative fold inductions and were not different than ATCC 3T3-L1 cells. Consistent responses were observed between cell lines for ligands of AR and ER, despite 80–100x greater expression of ERα in OP9 than 3T3-L1 cells. Unfortunately, it was beyond the scope of this manuscript to fully delineate changes in gene expression throughout differentiation as well as co-regulator expression across each cell line that may be required to fully describe the disparate results reported herein. Future research should attempt to determine the precise mechanism for the observed effects.

While PPARγ is often considered the only nuclear receptor whose activation is necessary and sufficient to initiate adipogenesis^32^, activation of LXR may regulate adipogenesis through up-regulation of PPARγ gene expression^33^,^34^, and activation of GR^35^, RXR^36^,^37^, and inhibition of TR^30^,^38^,^39^ and AR^40^,^41^ appear to help regulate the extent of differentiation and relative degree of lipid accumulation by maturing adipocytes. As such, while Zenbio 3T3-L1 cells exhibit less triglyceride accumulation through some pathways than ATCC 3T3-L1 cells, they also demonstrate greater cell proliferation and thus may represent the best option for identifying adipogenic chemicals able to act through one of these two mechanisms. Future studies should assess both of these mechanisms and relative contributions to identifying chemicals that can disrupt metabolism *in vivo*.

It is still unclear to what degree the wide heterogeneity in media additives may play in the degree of differentiation observed between studies, with widely divergent insulin, DEX, and other additives utilized between laboratories. Importantly, we did not include DEX in our differentiation cocktail; some studies have likewise opted to avoid using it^35^,^42^, while others utilize a 1,000-fold range of concentrations^22^,^43^,^44^,^45^. This is problematic, as endogenous glucocorticoid concentrations occur at a median of approximately 200 nM in children and adults^46^,^47^ and DEX is approximately 30-times more potent than endogenous glucocorticoids^48^. Relatively few studies have therefore utilized physiologically relevant concentrations of glucocorticoids and more commonly utilize μM concentrations that are likely 1,000-fold greater than endogenous levels. The addition of glucocorticoids to the differentiation cocktail provides an additional “priming” of preadipocytes and allows for greater subsequent accumulation of triglycerides^35^. By opting not to use DEX in our system, we allowed for the testing of DEX sensitivity directly between cell lines and also honed our chemicals to those able to stimulate adipogenesis progression without the influence of glucocorticoids. It is also unclear to what degree the frequency of media replacement influences adipogenic responses, as metabolism of contaminants has been previously demonstrated^43^. It is likely that these various methodological details contribute to some of the conflicting results reported. Standardization of protocols should be considered to bolster reproducibility and increase confidence in this body of work, while inter-lab comparison studies should be employed to adequately assess the impact on detection rates and prioritization.

The adipogenic activity of TBT has been well described both *in vitro*^43^,^49^,^50^ and *in vivo*^51^,^52^, and its dual agonist activities for PPAR and RXR have been well documented^49^,^53^; as such, it is a good test compound to evaluate adipogenesis and compare with LG100268 and RSG responses. Importantly, the triglyceride accumulation magnitude exhibited in ATCC 3T3-L1 cells is consistent with previous studies that also utilized ATCC-sourced 3T3-L1 cells^43^,^50^,^54^. Greater triglyceride accumulation was observed in OP9 cells, and a minimal response was observed in Zenbio 3T3-L1 cells. Significant triglyceride accumulation and cell proliferative responses were observed across cell lines, suggesting each would have successfully identified this chemical for further investigation. Relative responses to the RXR ligand LG100268 were similar to TBT, and RXRβ expression was significantly greater in ATCC 3T3-L1 cells, suggesting that RXR activity is the likely mechanism for the varying TBT activities. Interestingly, though ten or fourteen days was required for maximal differentiation of most chemicals in 3T3-L1 cells, TBT induced the greatest triglyceride accumulation at seven days in Zenbio and OP9 cells and greatest proliferation at seven days in all cell lines, complicating the selection of universal protocols to screen unknown chemicals.

Notable differences were also observed in the comparison of BPA and its structural analogues. Increased cell proliferation was observed for BPA, TBBPA, TCBPA, and BPAF in both ATCC 3T3-L1 cells and OP9 cells but not Zenbio 3T3 L1 cells. Despite this, all four increased triglyceride accumulation in both 3T3-L1 sourced cells, though BPA and BPAF were inactive in OP9 cells. TBBPA and TCBPA exhibited the greatest activity in all cells, perhaps due to their greater relative antagonism of the farnesoid X receptor and/or activation of PPARγ; both mechanisms contributing to adipogenesis^55^,^56^. TCBPA was generally the most active chemical, potentially due to greater inhibition of TR or activation of PPARγ^57^,^58^. TBBPA, TCBPA, and BPA have been previously reported to increase triglyceride accumulation and/or cell proliferation in 3T3-L1 cells, though the maximal rosiglitazone response is often not provided for ease of translation to relative effect and subsequent comparison to other studies^35^,^45^,^57^,^59^. BPA has been shown previously to increase adipogenic gene expression (aP2) through a non-classical ER-mediated mechanism^60^, suggesting that these mechanisms could account for the disparate response. To the best of our knowledge, BPAF has not previously been reported as an adipogenic chemical.

Lastly, T0070907 and GW9662 exhibited more potent inhibition of triglyceride accumulation via PPARγ antagonism in Zenbio 3T3-L1 and OP9 cells, though decreased DNA content was only observed in the OP9 cells. GW9662 has been previously reported to lack proper inhibition in ATCC 3T3-L1 cells due to a short half-life relative to rosiglitazone, which may have led to the less potent inhibition we observed in these cells^43^. As PPARα and PPARγ are more highly expressed in OP9 cells, this may help explain the greater response observed in these cells. Generally, lower inhibitory potencies were observed with longer induction times in OP9 cells and no differences were apparent in 3T3-L1 cells; this may suggest that antagonists could be assessed with shorter exposures without a subsequent loss in sensitivity. Future studies should investigate chemical half-life in each cell line to determine the potential impact of this variable on the results described herein.

Striking differences were observed based on the cell culture plastic utilized. All plates were 96-well black polystyrene plates with clear bottoms, though each was coated with proprietary company-specific tissue culture coatings. Both Labnet and Brandtech plates resulted in approximately half of the maximal fold induction relative to vehicle of Greiner plates, impacting the potencies. Notable cytotoxicity or decreased proliferative response was observed with increasing concentrations of RSG in the Brandtech plates only, suggesting that utilizing specific tissue culture products may significantly impact the ability to detect chemicals that act via increased pre-adipocyte proliferation. Unfortunately, due to the proprietary nature of the coatings, the mechanism for these effects cannot be determined. We suspect that given the diversity of receptor-mediated pro-adipogenic pathways, it is possible that some of these plates or coatings contain impurities or constituent chemicals that interfere with the differentiation process. Alternatively, recent research suggested a potential mechanism for the observed effects; certain plate coatings suppress protein adsorption, leading to suppressed actin assembly and integrin signaling in 3T3-L1 cells. This altered cell signaling resulted in decreased cell growth and subsequent suppression of Rho-associated kinase (ROCK) and TAZ, which are activated during cell spreading. Suppression of these factors results in activation of PPARγ and promotion of adipogenesis, suggesting the plate coatings we utilized could have impacted adipogenesis through a similar mechanism^61^.

Utilizing OP9 cells offers several advantages, notably that they more readily differentiate in shorter time frames^8^. Longer induction times did not improve sensitivity, and they do not require growth arrest prior to differentiation, as do 3T3-L1 cells. This reduced timeline contributes to lower potential error and cost than in 3T3-L1 cells. Despite these strengths, two of the four phenols tested (BPA and BPAF) were inactive in OP9 cells, despite all being active in 3T3-L1 cells. BPA has been previously demonstrated to act as an obesogen *in vivo*^62^,^63^. BPAF has, to the best of our knowledge, not been demonstrated as an obesogen *in vitro* or *in vivo,* but does act as a PPARγ agonist and AR antagonist^64^,^65^. As such, given the mechanism of action for BPA/BPAF and the divergent adipogenic/lipogenic pathways in these cells relative to 3T3-L1, these are likely false negatives for OP9. While this is a small set of chemicals tested, this false negative rate is inappropriately high to make this model realistic as a screening tool, particularly considering the lower relative responses to LXR, GR, and TR-driven triglyceride accumulation.

A greater concern is the heterogeneity of response between the different cell sources of 3T3-L1 cells. In 2012, Zebisch *et al*. reported that ATCC maintained two 3T3-L1 lots, passage unknown (u) + 4 and u + 12^27^. Currently, ATCC maintains five lots (lot# 2268173, passage u + 8, frozen 2002; lot# 62996847, u + 14, 2014; lot# 62485415, passage u + 14, 2015; lot# 63343749, u + 13, 2015; lot# 61194648, u + 14, unlisted date). Certificates of analysis list that adipocytes form between 7–10 days for these lots, demonstrating that ATCC has not maintained continuity of response between lots of 3T3-L1 cells. Interestingly, a recent publication highlighted phenotypic and cellular heterogeneity in a single batch of MCF-7 cells distributed by ATCC^66^, suggesting this may be a common concern. Zenbio, Inc. (Research Triangle Park, NC) recently began providing 3T3-L1 cells at passage 8 directly from the source laboratory^67^,^68^. As such, the provenance of these cells is established and may provide more consistent responses than the heterogeneous population otherwise available. As we reported herein, we found the ATCC 3T3-L1 cells differentiate inconsistently, contributing to greater standard error and lower resultant sensitivity. Zenbio 3T3-L1 cells, while they sometimes exhibited slightly lower sensitivity, also exhibited lower replicate error and greater reproducibility. Given the differentiation responses between the cell lines, the ATCC cells may reflect a pre-adipocyte cell line slightly further along the differentiation pathway than the Zenbio cells, though not as far as the OP9 cells.

Additional work is needed to clarify the mechanisms of the increased cell proliferation observed between cell lines and chemicals. Adipocytes, as terminally differentiated cells, do not proliferate^69^, though pre-adipocytes proliferate in response to EDCs^70^,^71^. However, despite identical pre-differentiation windows, differences were noted herein in DNA content between different induction times (7, 10, 14 days; Figs 1, 2, 3, 4). Several possibilities could explain this. Perhaps most likely, the adipogenic cocktail does not induce all of the cells present to differentiate into mature adipocytes; as such, there would remain a pool of preadipocytes that would contribute to larger cell numbers with longer induction periods. There is also potential that adipocytes are not terminally differentiated and retain some ability to proliferate, as has been shown for cardiomyocytes and some other cell types^72^. As has been previously noted, the ability for mature adipocytes to proliferate is contested^73^, as several studies have demonstrated the ability of mature adipocytes to proliferate *in vitro*^74^ or permanent modulation of adult adipocyte number following a transient exposure^71^,^75^. As such, tritiated thymidine or 5-bromo-2′-deoxyuridine incorporation experiments should be considered throughout differentiation to determine when the cells are proliferating and whether there are differences between these cell lines.

In conclusion, we report herein that numerous variables can significantly impact the detection of adipogenic chemicals. It is clear based on these results that greater protocol transparency and homogeneity is required in assessing adipogenesis *in vitro,* as has been previously suggested^22^. Most importantly, comparing adipogenic responses between studies is nearly impossible when complete dose responses of reference compounds are not included. Despite this, most studies present either one positive control concentration or only present fold induction relative to vehicle; this fails to demonstrate maximal response or sensitivity of the cells and provides insufficient data for subsequent replication. Cell source and differentiation protocols must be clearly defined, as this can contribute to a wide degree of variation. It is also clear that both triglyceride accumulation and cell proliferation should be assessed, as chemicals acting through one mechanism or the other may be otherwise missed. While the majority of laboratories appear to utilize the ATCC 3T3-L1 cells, the provenance of these cells is questionable and discordant responses are observed between these lots and in relation to the originally isolated 3T3-L1 cells (Zenbio).

## Materials and Methods

### Chemicals

Chemicals were purchased as follows: RSG (Sigma cat # R2408, ≥98%), tributyltin chloride (Aldrich cat # T50202, 96%), T0070907 (Tocris cat # 2301, >99%), GW9662 (Sigma cat # M6191, >98%), BPA (Sigma cat # 239658, >99%), TBBPA (Aldrich cat # 25,759–1, >99%), TCBPA (Aldrich cat # 330396, >99%), BPAF (TCI America cat # T0062, >99%), GW3965 (Sigma cat #G6295, ≥98%), E2 (Sigma cat # E8875, ≥98%), flutamide (Sigma cat # F9397, ≥99%), 1–850 (Millipore cat # 609315, ≥98%), DEX (Sigma cat # D1756, ≥98%), and LG100268 (Sigma cat # SML0279, ≥98%). Stock solutions were prepared in 100% DMSO (Sigma cat # D2650) and stored at −20 °C between uses.

### Cell Culture

OP9 cells were obtained from the ATCC (cat# CRL-2749, lot# 3984779) through a Material Transfer Agreement with the Duke Cancer Institute Cell Culture Facility. OP9 cells were maintained in Minimum Essential Medium (MEM) alpha without ribonucleosides/deoxyribonucleosides (Gibco cat# 12561) supplemented with 20% fetal bovine serum and 1% penicillin and streptomycin, as described previously^7^. OP9 cells were routinely passaged upon reaching confluency.

3T3-L1 cells were obtained from two sources: one vial was obtained from the ATCC (cat# CL-173, lot# 2268173) through the Duke Cell Culture Facility, and the other was purchased from Zenbio, Inc. (cat# SP-L1-F, lot# 3T3062104; Research Triangle Park, NC). 3T3-L1 cells were maintained in Dulbecco’s Modified Eagle Medium – High Glucose (Gibco cat# 11995) supplemented with 10% bovine calf serum and 1% penicillin and streptomycin, as described previously^22^,^68^. To prevent the loss of contact inhibition over time, cells were passaged upon reaching 60–80% confluency and maintained in a sub-confluent state until prepared for differentiation.

3T3-L1 cells were used within six passages of thawing and OP9 within ten; no significant decreases were observed in differentiation capability over that time (data not shown).

### Differentiation Induction and Maintenance

To induce differentiation, cells were seeded into 96-well black clear-bottom tissue culture plates (Greiner cat # 655090) at approximately 30,000 cells per well and grown to confluence in respective growth medium. Upon reaching confluency, cells were cultured for a further 48 hours in growth media to initiate growth arrest. After this window, to initiate clonal expansion and differentiation, media was replaced with test chemical/control dilution series (0.1 nM to 1.0 μM for RSG, TBT; 1 nM to 10 μM all other chemicals) in a 0.1% DMSO vehicle diluted in differentiation media (base media for OP9 and 3T3-L1 described above, supplemented with 10% fetal bovine serum, 1% penicillin and streptomycin, 1.0 μg/mL human insulin, and 0.5 mM 3-isobutyl-1-methylxanthine). For antagonist testing, cells were co-exposed to a test chemical dilution series (1 nM to 10 μM) as well as EC_50_ concentrations of rosiglitazone in each cell line: 9.0 nM, 30 nM, and 15 nM for ATCC 3T3-L1, Zenbio 3T3-L1, and OP9 cells, respectively. Following 48 hours of exposure, media was replaced with test chemicals and controls diluted in adipocyte maintenance media (differentiation media without 3-isobutyl-1-methylxanthine). This maintenance media (along with test chemicals and dilutions) was refreshed every 2–3 days until plates were assayed.

### Lipid and DNA staining protocols

Plates were assayed for lipid accumulation and DNA content on day seven and ten after induction of differentiation for OP9 and 3T3-L1 cells, respectively. Media was removed from wells and cells rinsed gently with Dulbecco’s phosphate-buffered saline with calcium and magnesium (DPBS; Gibco cat # 14040) before replacing with 200 μL DPBS with NucBlue added (DNA dye), as per manufacturer’s instructions (Thermo cat # R37605) and incubated at room temperature for twenty minutes. For lipid accumulation, 5 μL of AdipoRed (Lonza cat # PT-7009) was then added to each well and protected from light for fifteen minutes at ambient temperature. Plates were read using a Molecular Devices SpectraMax M5 fluorimeter with excitation at 485 nm and emission at 572 nm for AdipoRed fluorescence and excitation at 360 nm and emission at 460 nm for NucBlue fluorescence. Percent activities were calculated relative to the maximal fold induction of rosiglitazone over differentiated vehicle controls (0.1% DMSO) within each assay, after subtracting raw fluorescence units determined from cell-free wells to account for background fluorescence. Percent inhibition was calculated as the percent reduction in fluorescence relative to the half maximal response of rosiglitazone (approximately 30 nM). DNA content was calculated as a percent change from vehicle control at each test chemical concentration. Potencies were determined based on EC_20_/EC_50_ values (concentration of test chemical that exhibits 20% or 50% of its maximal activity) for agonists and IC_20_/IC_50_ values (concentration of test chemical that inhibits 20% or 50% of half-maximal positive control response) for antagonists. Efficacies were determined based on percent activities relative to the maximal rosiglitazone response.

### Experimental protocols

The above protocols were followed for the majority of work performed herein. For some experiments, various differentiation windows were used; 48 hours incubation post-confluence was used for all experiments (although OP9 cells do not require this window^8^), but induction and differentiation times varied by experiment. Unless otherwise specified, ATCC and Zenbio 3T3-L1 cells were differentiated/induced for 10 days (2 days differentiation cocktail, 8 days maintenance) and OP9 cells for 7 (2 days cocktail, 5 days maintenance). Induction time, including the two-day differentiation cocktail, was tested at 7, 10, or 14 days to span the range of induction periods used by other researchers.

In another set of experiments, various brands of polystyrene 96-well tissue culture plates were used. Unless otherwise specified, the default tissue culture plate used was the Greiner Bio-One CELLSTAR™ black clear-bottom tissue culture plate (VWR cat # 82050–748). Other 96-well plates evaluated included the Brandtech cellGrade™ (MidSci cat # 781971) and the Labnet International Krystal™ 2000 (Genesee cat # 33–615X) black clear-bottom tissue culture plates, where specified.

### Nuclear Receptor Gene Expression

Cultures of confluent un-differentiated ATCC 3T3-L1, Zenbio 3T3-L1 and OP9 cells were generated from frozen stocks. Total RNA was extracted and genomic DNA was removed using the RNeasy Plus Mini Kit (Qiagen, Valencia, CA) from three replicates of each cell line. cDNA was prepared from total RNA using the GoScript™ Reverse Transcription System (Promega), with a 1:1 mixture of random and Oligo (dT)_15_ primers. All qPCR reactions were performed using the GoTaq^®^ qPCR Master Mix System (Promega). Validated primers were purchased from Qiagen or synthesized by Integrated DNA Technologies (Coralville, IA; Table S2) and were used at 100 nM. qPCR reactions were performed using a 7300 Fast Real-Time PCR System (Applied Biosystems, Carlsbad, CA): Hot-Start activation at 95 °C for 10 min, 40 cycles of denaturation (95 °C for 15 sec) and annealing/extension (55 °C for 60 sec). Relative gene expression was determined using the Pfaffl method to account for differential primer efficiencies^76^. The geometric mean of the Cqs value for 18 s ribosomal RNA (*Rn18s*), beta-2-microglobulin (*B2m*) and beta-actin (*Atcb*) was used for normalization. The average Cq value for three independent 3T3-Swiss Albino cultures (ATCC, CCL-92) was used as the reference point, and the data are reported as “Relative Expression”. Significant differences between cell lines was tested using a one-way ANOVA and Tukey multiple comparisons test in GraphPad Prism 6.0 (GraphPad Software, Inc.) with differences considered significant at p < 0.05.

### Statistical Analysis

Data are presented as means ± SE from four technical replicates of three independent experiments. Linear mixed models were used to analyze the results from the three biological replicate assays, and incorporated random effects to account for dependence among quadruplicate technical replicates. Post-test comparison between treatment groups was performed between groups using least-square means to determine 95% confidence intervals and the Tukey-Kramer multiple comparison test with differences considered statistically significant at p < 0.05 to determine differences between treatment groups and from vehicle control. Cell proliferation results were log transformed for normal distributions and adjusted means back-transformed for presentation. Proc GLIMMIX in SAS 9.4 (SAS Inc.) was used for this analysis. EC/IC50 values were estimated using curves generated from raw fluoresence data using a 4-parameter variable-slope Hill model in GraphPad Prism 6.0.

## Additional Information

**How to cite this article**: Kassotis, C. D. *et al*. Characterization of Adipogenic Chemicals in Three Different Cell Culture Systems: Implications for Reproducibility Based on Cell Source and Handling. *Sci. Rep.* **7**, 42104; doi: 10.1038/srep42104 (2017).

**Publisher's note:** Springer Nature remains neutral with regard to jurisdictional claims in published maps and institutional affiliations.

## Supplementary Material

Supplemental Information

## Figures and Tables

**Table 1:** Comparison of Adipogenic Activity by Test Compounds.

Chemical	Cell Line	EC20/IC20 (μM)	EC50/IC50 (μM)	Efficacy
**Rosiglitazone**	ATCC 3T3-L1	0.002 ± 3.1E-4	0.009 ± 2.2E-4	100.0%
	Zenbio 3T3-L1	0.01 ± 0.9E-5	0.03 ± 1.1E-4	100.0%
	OP9	0.007 ± 1.1E-4	0.02 ± 1.2E-4	100.0%
**TBT**	ATCC 3T3-L1	0.001 ± 1.5E-4	0.006 ± 5.6E-4	74.5% ± 9.6%
	Zenbio 3T3-L1	0.001 ± 1.1E-4	0.003 ± 2.1E-4	15.6% ± 3.3%
	OP9	0.009 ± 1.5E-4	0.02 ± 3.8E-3	132.3% ± 17.6%
**T0070907**	ATCC 3T3-L1	1.67 ± 1.3E-4	3.06 ± 2.2E-4	91.9% ± 2.9%
	Zenbio 3T3-L1	0.16 ± 1.2E-5	1.28 ± 4.8E-5	96.3% ± 0.3%
	OP9	0.62 ± 3.3E-5	1.53 ± 9.1E-5	100.0% ± 1.9%
**GW9662**	ATCC 3T3-L1	1.17 ± 0.13	2.52 ± 0.21	83.4% ± 10.0%
	Zenbio 3T3-L1	1.25 ± 0.04	2.28 ± 0.09	81.9% ± 5.7%
	OP9	0.04 ± 3.9E-4	0.70 ± 1.1E-3	100.0% ± 0.8%
**BPA**	ATCC 3T3-L1	0.58 ± 2.2E-4	2.15 ± 4.6E-3	23.0% ± 2.9%
	Zenbio 3T3-L1	N/A	N/A	2.9% ± 0.3%
	OP9	N/A	N/A	N/A
**TBBPA**	ATCC 3T3-L1	0.27 ± 1.8E-4	1.68 ± 2.5E-4	45.7% ± 3.3%
	Zenbio 3T3-L1	0.80 ± 1.2E-4	2.89 ± 2.2E-4	16.4% ± 3.7%
	OP9	1.33 ± 4.1E-5	3.44 ± 9.2E-4	28.4% ± 0.8%
**TCBPA**	ATCC 3T3-L1	0.88 ± 2.4E-4	2.73 ± 0.27	46.3% ± 2.6%
	Zenbio 3T3-L1	1.18 ± 0.09	3.23 ± 0.14	34.9% ± 15.4%
	OP9	0.85 ± 4.4E-5	2.83 ± 0.06	19.7% ± 0.6%
**BPAF**	ATCC 3T3-L1	0.57 ± 0.06	2.10 ± 0.19	48.5% ± 10.7%
	Zenbio 3T3-L1	1.25 ± 0.17	3.33 ± 0.08	33.3% ± 12.6%
	OP9	N/A	N/A	N/A

Values provided as the mean of three (or more) replicate assays ± standard error of the mean. Efficacy provided as the maximum relative percent agonist activity relative to the maximum rosiglitazone response or the maximum percent inhibition relative to the added agonist response for antagonists T0070907 and GW9662.

EC_20_ = concentration of test chemical (agonist) at which it exhibits 20% of its maximal activity.

EC_50_ = concentration of test chemical (agonist) at which it exhibits 50% of its maximal activity.

IC_20_ = concentration of test chemical (antagonist) at which it inhibits 20% of half-maximal rosiglitazone response.

IC_50_ = concentration of test chemical (antagonist) at which it inhibits 50% of half-maximal rosiglitazone response.

Rosiglitazone = PPARy positive control agonist, TBT = tributyltin chloride, T0070907 = adipogenesis antagonist control, GW9662 = PPARy antagonist control, BPA = bisphenol A, TBBPA = tetrabromobisphenol A, TCBPA = tetrachlorobisphenol A, BPAF = hexafluorobisphenol A.
