# Supplementary material for: Characterization of Adipogenic Chemicals in Three Different Cell Culture Systems: Implications for Reproducibility Based on Cell Source and Handling
Source: Sci Rep. 2017 Feb 8;7:42104. doi: 10.1038/srep42104 (PMC5296734; doi:10.1038/srep42104)
Supplement: Supplemental Information [file srep42104-s1.pdf]

Supplemental Information

Characterization of Adipogenic Chemicals in Three Different Cell Culture Systems: Implications for Reproducibility and Variations Based on Cell Source and Handling

Christopher D. Kassotis[1], Lauren Masse[2], Stephanie Kim[2], Jennifer J. Schlezinger[2], Thomas F. Webster[2], and Heather M. Stapleton[1,*]

[1] Nicholas School of the Environment, Duke University, Durham, NC 27708

[2] Department of Environmental Health, Boston University School of Public Health, Boston, MA 02118

*corresponding author: heather.stapleton@duke.edu

Contents

Table S1, Receptor ligand activities vary between cell lines and sources Page S2

Table S2, List of primers used for qPCR Page S3

Figure S1, Representative imaging of seven-day differentiated adipocytes Page S5

Figure S2, Representative imaging of ten-day differentiated adipocytes Page S6

Figure S3, Representative imaging of fourteen-day differentiated adipocytes Page S7

Figure S4, Positive controls induce varied adipogenic activities between cell lines Page S8

Figure S5, Negative controls induce varied adipogenic activities between cell lines Page S9

Table S1. Receptor ligand activities vary between cell lines and sources.

Receptor	ATCC 3T3-L1	Zenbio 3T3-L1	OP9
PPARγ agonism	+++	+++	+++
LXR agonism	+	+	-
GR agonism	++++*	++++*	+
RXR agonism	++	+	++++
TR antagonism	+	++	-
TR agonism	-	-	-
AR antagonism	+	+	+
ER agonism	-	-	-

Descriptive overview of receptor pathway testing using ligands for specific nuclear receptors involved in adipogenesis. ATCC 3T3-L1, Zenbio 3T3-L1, and OP9 cells were differentiated as described in Methods and assessed for adipocyte differentiation (Nile Red staining of lipid accumulation) and cell proliferation (Hoechst staining) following seven days (OP9) or ten days (3T3-L1) of treatment with positive control chemicals. Testing of PPARγ utilized rosiglitazone, LXR used GW3965, GR used dexamethasone, RXR used LG100268, TR antagonism used 1-850, TR agonism used triiodothyronine, AR used flutamide, and ER used 17β-estradiol.

PPARγ = peroxisome proliferator activated receptor gamma, LXR = liver X receptor, GR = glucocorticoid receptor, RXR = retinoid X receptor, TR = thyroid receptor, AR = androgen receptor, ER = estrogen receptor.

Activities defined as follows: - = 0-7% relative triglyceride accumulation, + = 8-25%, ++ = 26-50%, +++ = 51-75%, ++++ = 76-100%.

** = superinduction: Zenbio and ATCC 3T3-L1 cells exhibited approximately 300% and 150% relative triglyceride accumulation, respectively.*

Table S2. List of primers used for qPCR.

Primer	Manufacturer	Sequence/Catalog #
Actb	IDT	F: CTCTGGCTCCTAGCACCATGAAGA<br>R: GTAAAACGCAGCTCAGTAACAGTCCG
B2m	IDT	F: CTGCTACGTAACACAGTTCCACCC<br>R: CATGATGCTTGATCACATGTCTCG
Esr1 (Nr3a1)	Qiagen	Cat # QT01075641
Esr2 (Nr3a2)	Qiagen	Cat # QT01761879
Gcr (Nr3c1)	Qiagen	Cat # QT01757735
Lxra (Nr1h3)	IDT	F: GAGTTGTGGAAGACAGAACCTCAA<br>R: GGGCATCCTGGCTTCCTC
Lxrb (Nr1h2)	IDT	F: CCCCACAAGTTCTCTGGACAC<br>R: TGGCGGAGGTACTGGGC
Nurr1 (Nr4a2)	Qiagen	Cat # QT00106407
Ppara (Nr1c1)	IDT	F: GCGTACGGCAATGGCTTTAT<br>R: GAACGGCTTCCTCAGGTTCTT
Ppard (Nr1c2)	Qiagen	Cat # QT00166292
Pparg (Nr1c3)	IDT	F: TGGGTGAAACTCTGGGAGATTC<br>R: AATTTCTTGTGAAGTGCTCATAGGC
Rara (Nr1b1)	Qiagen	Cat # QT00125958
Rarb (Nr1b2)	Qiagen	Cat # QT00151956
Rarg (Nr1b3)	Qiagen	Cat # QT01554322
Rn18s	IDT	F: GTAACCCGTTGAACCCCATT<br>R: CCATCCAATCGGTAGTAGCG
Rxra (Nr2b1)	IDT	F: TACCCACCACACCCACATTG<br>R: GCCTAGTGGCGGCTTGATATC
Rxrb (Nr2b2)	IDT	F: GAGCTCCTCATTGCGTCCTT<br>R: GCGGAATGGGCTGAGTTTC

Rxrg (Nr2b3)	IDT	F: CAGGTCTGCCTGGGATTGGA<br>R: GTTGAGTTCTCCACGTTCATG

Primers used, manufacturers, and sequence or catalog numbers for all genes tested by qPCR to assess adipogenic pathways.

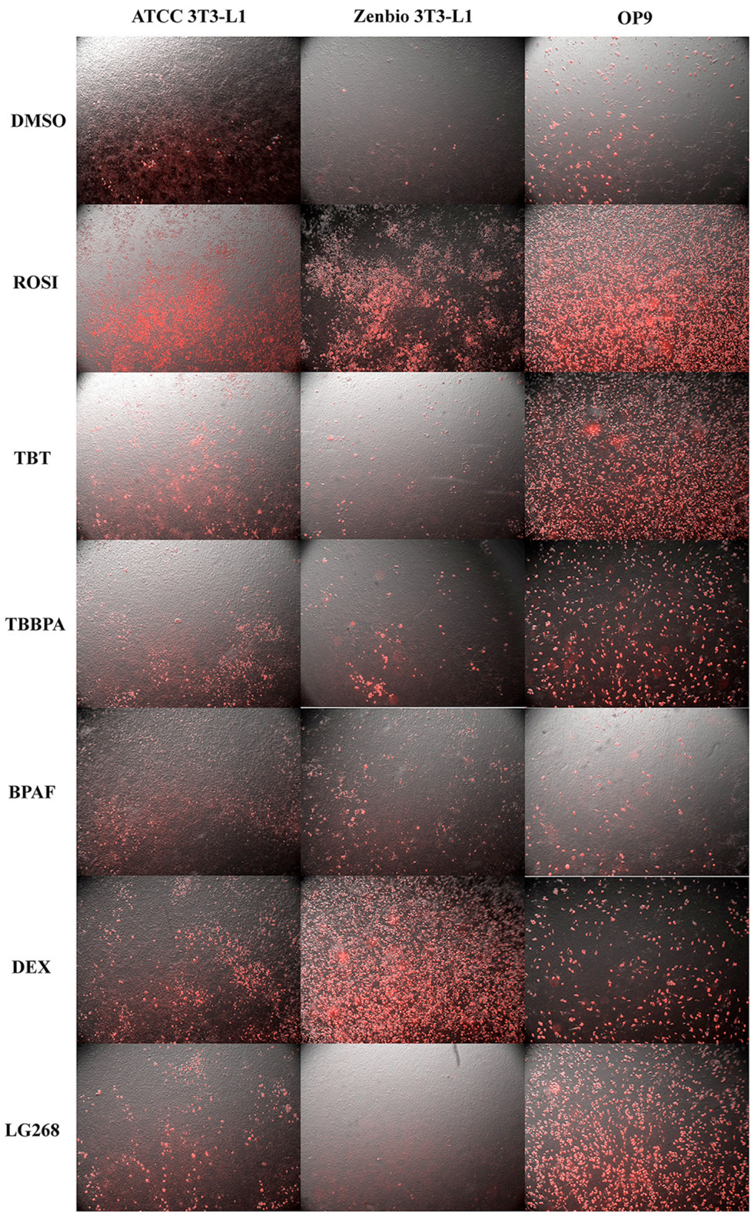

Supplemental Figure 1: **Representative imaging of seven-day differentiated adipocytes.** *ATCC 3T3-L1, Zenbio 3T3-L1, and OP9 cells were differentiated as described in Methods in 12-well Greiner Bio-One plates and assessed for adipocyte differentiation (Nile Red staining of lipid accumulation) following seven days treatment with positive control chemicals. Differentiated cells were imaged at 40x magnification using a Zeiss Lumar fluorescent stereoscope in both bright field and Rhodamine (red) imaging mode. Fluorescent lipid staining images were merged into bright field images for final presentation. Representative images provided of triglyceride accumulation in ATCC 3T3-L1 cells (column 1), Zenbio 3T3-L1 cells (column 2), and OP9 cells (column 3). Concentrations of test ligands selected to be the lowest concentration that exhibited maximal differentiation across most or all cell lines.*
VEH = 0.1% DMSO vehicle control, RSG = 100 nM rosiglitazone, TBT = 100 nM tributyltin chloride, TBBPA = 10 µM tetrabrominated bisphenol A, BPAF = µM bisphenol AF, DEX = 10 nM dexamethasone, LG268 = 10 nM LG100268.

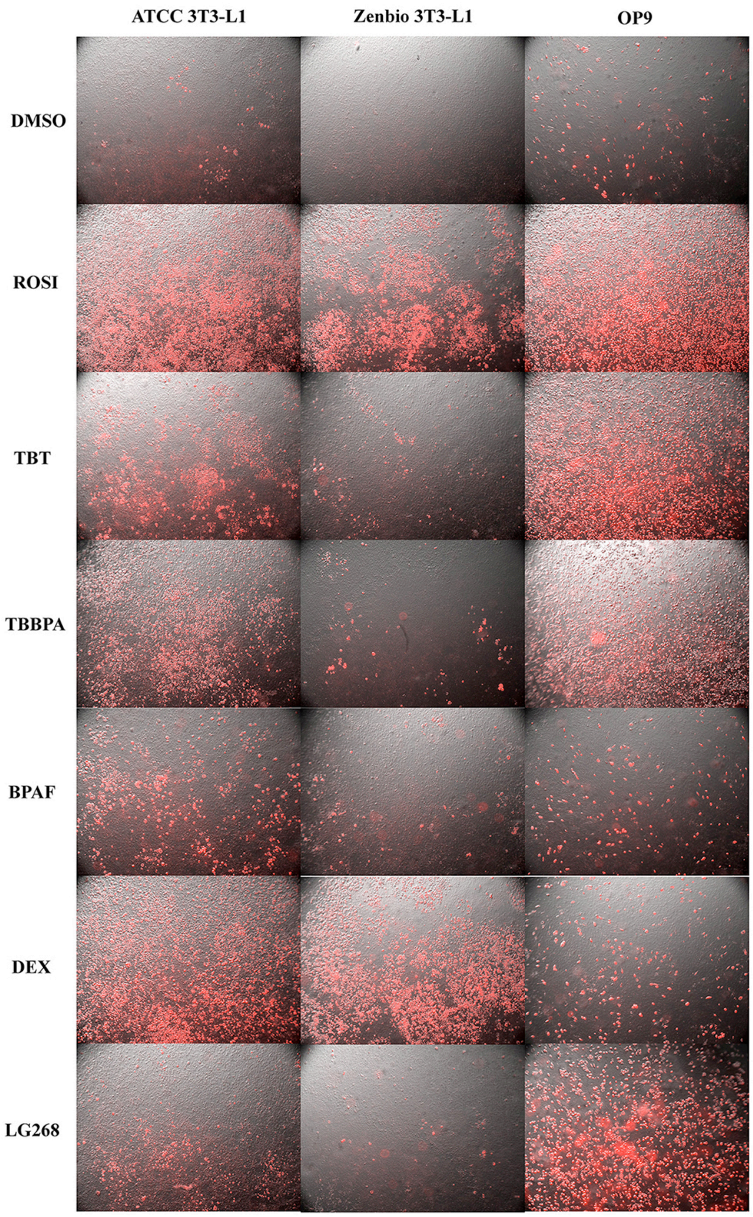

Supplemental Figure 2: **Representative imaging of ten-day differentiated adipocytes.** *ATCC 3T3-L1, Zenbio 3T3-L1, and OP9 cells were differentiated as described in Methods in 12-well Greiner Bio-One plates and assessed for adipocyte differentiation (Nile Red staining of lipid accumulation) following ten days treatment with positive control chemicals. Differentiated cells were imaged at 40x magnification using a Zeiss Lumar fluorescent stereoscope in both bright field and Rhodamine (red) imaging mode. Fluorescent lipid staining images were merged into bright field images for final presentation. Representative images provided of triglyceride accumulation in ATCC 3T3-L1 cells (column 1), Zenbio 3T3-L1 cells (column 2), and OP9 cells (column 3). Concentrations of test ligands selected to be the lowest concentration that exhibited maximal differentiation across most or all cell lines.*
VEH = 0.1% DMSO vehicle control, RSG = 100 nM rosiglitazone, TBT = 100 nM tributyltin chloride, TBBPA = 10 µM tetrabrominated bisphenol A, BPAF = µM bisphenol AF, DEX = 10 nM dexamethasone, LG268 = 10 nM LG100268.

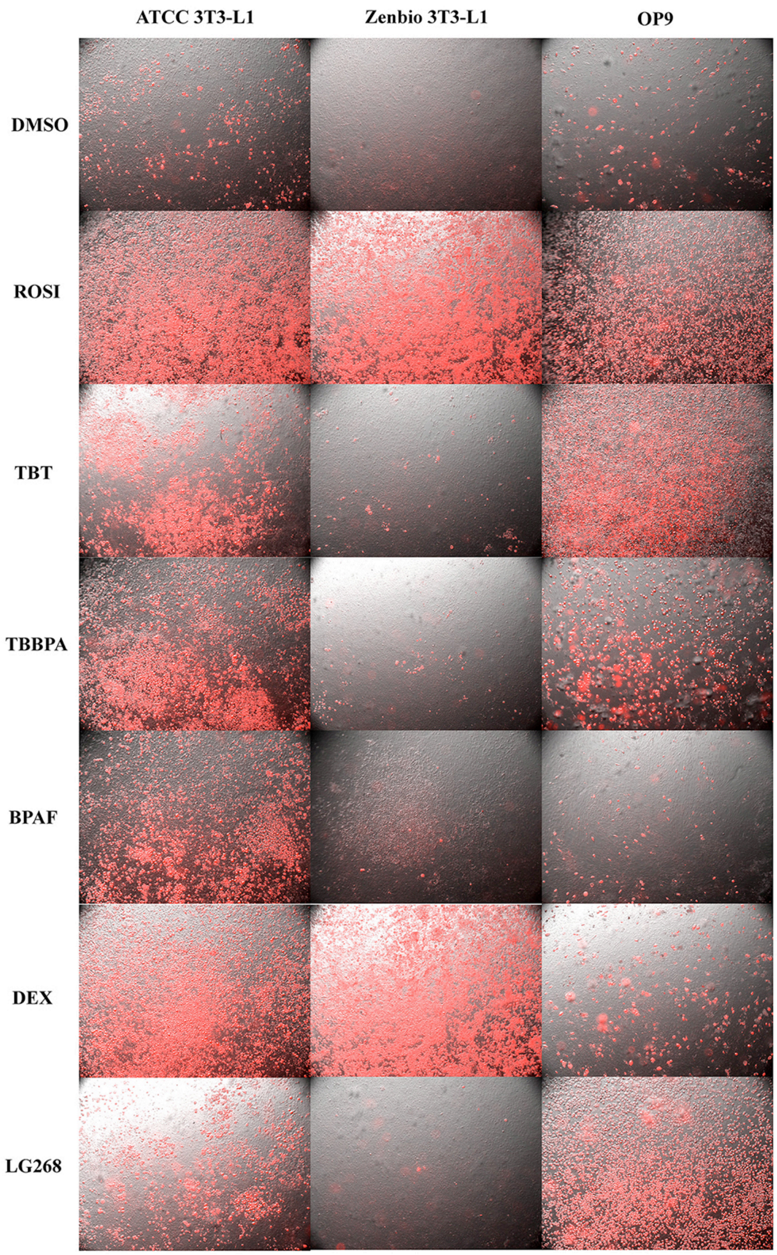

Supplemental Figure 3: **Representative imaging of fourteen-day differentiated adipocytes.** *ATCC 3T3-L1, Zenbio 3T3-L1, and OP9 cells were differentiated as described in Methods in 12-well Greiner Bio-One plates and assessed for adipocyte differentiation (Nile Red staining of lipid accumulation) following fourteen days treatment with positive control chemicals. Differentiated cells were imaged at 40x magnification using a Zeiss Lumar fluorescent stereoscope in both bright field and Rhodamine (red) imaging mode. Fluorescent lipid staining images were merged into bright field images for final presentation. Representative images provided of triglyceride accumulation in ATCC 3T3-L1 cells (column 1), Zenbio 3T3-L1 cells (column 2), and OP9 cells (column 3). Concentrations of test ligands selected to be the lowest concentration that exhibited maximal differentiation across most or all cell lines.*
VEH = 0.1% DMSO vehicle control, RSG = 100 nM rosiglitazone, TBT = 100 nM tributyltin chloride, TBBPA = 10 μM tetrabrominated bisphenol A, BPAF = μM bisphenol AF, DEX = 10 nM dexamethasone, LG268 = 10 nM LG100268.

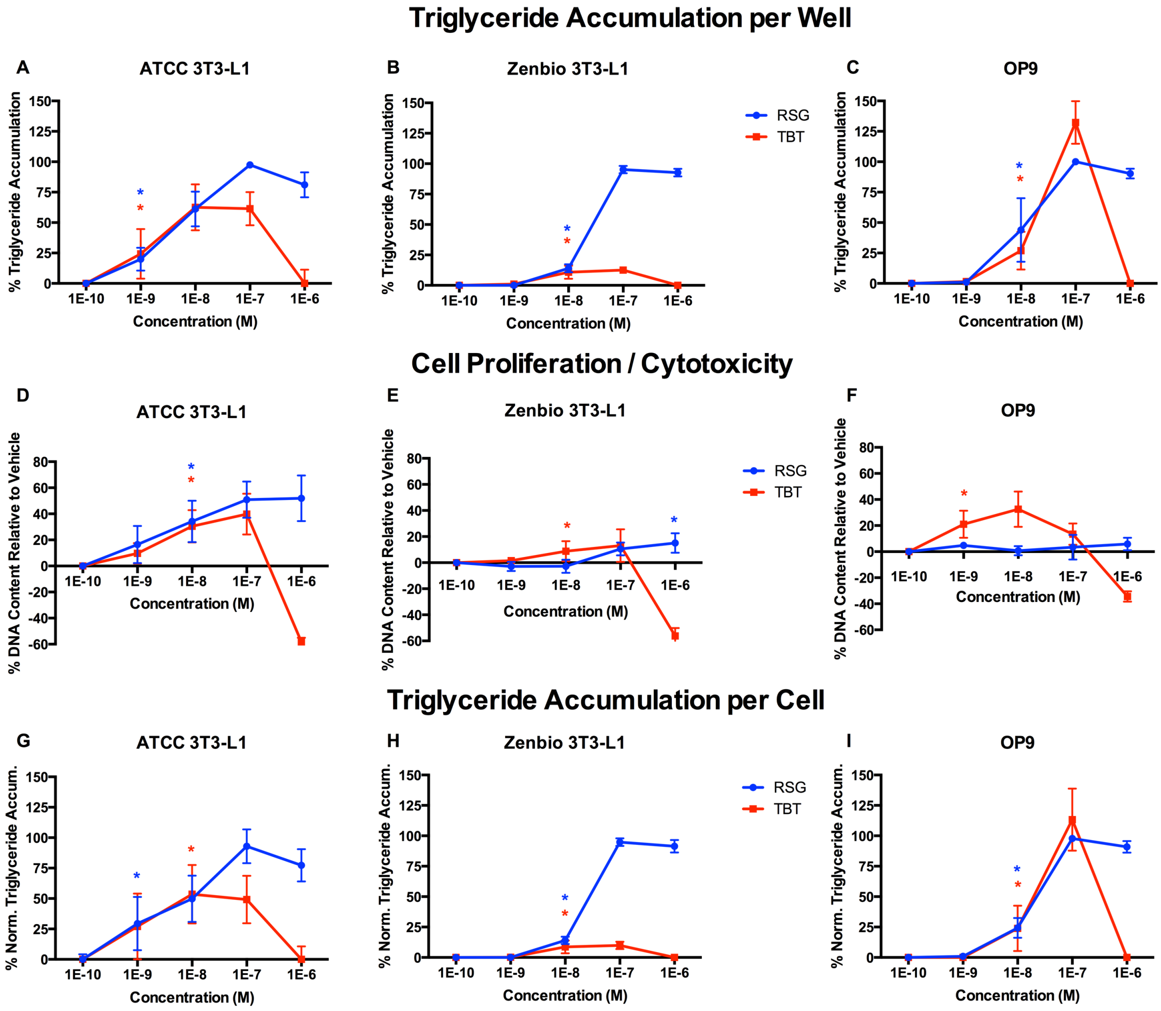

Supplemental Figure 4: **Positive Controls Induce Varied Adipogenic Activities Between Cell Lines.** *ATCC 3T3-L1, Zenbio 3T3-L1, and OP9 cells were differentiated as described in Methods and assessed for adipocyte differentiation (Nile Red staining of lipid accumulation) and cell proliferation (Hoechst staining) following seven days (OP9) or ten days (3T3-L1) of treatment with positive control chemicals. Percent raw triglyceride accumulation per well for rosiglitazone (RSG) and tributyltin chloride (TBT) in ATCC 3T3-L1 cells (A), Zenbio 3T3-L1 cells (B), and OP9 cells (C). Increase (cell proliferation) or decrease (potential cytotoxicity) in DNA content relative to vehicle control for test chemicals in ATCC 3T3-L1 cells (D), Zenbio 3T3-L1 cells (E), and OP9 cells (F). Percent normalized triglyceride accumulation per cell (normalized to DNA content) for test chemicals in ATCC 3T3-L1 cells (G), Zenbio 3T3-L1 cells (H), and OP9 cells (I). TBT response is provided as relative activity to maximal rosiglitazone. Data presented as mean ± SE from three independent experiments. * indicates lowest concentration with significant increase in response over vehicle control, $p<0.05$, as per linear mixed effect model in SAS.*

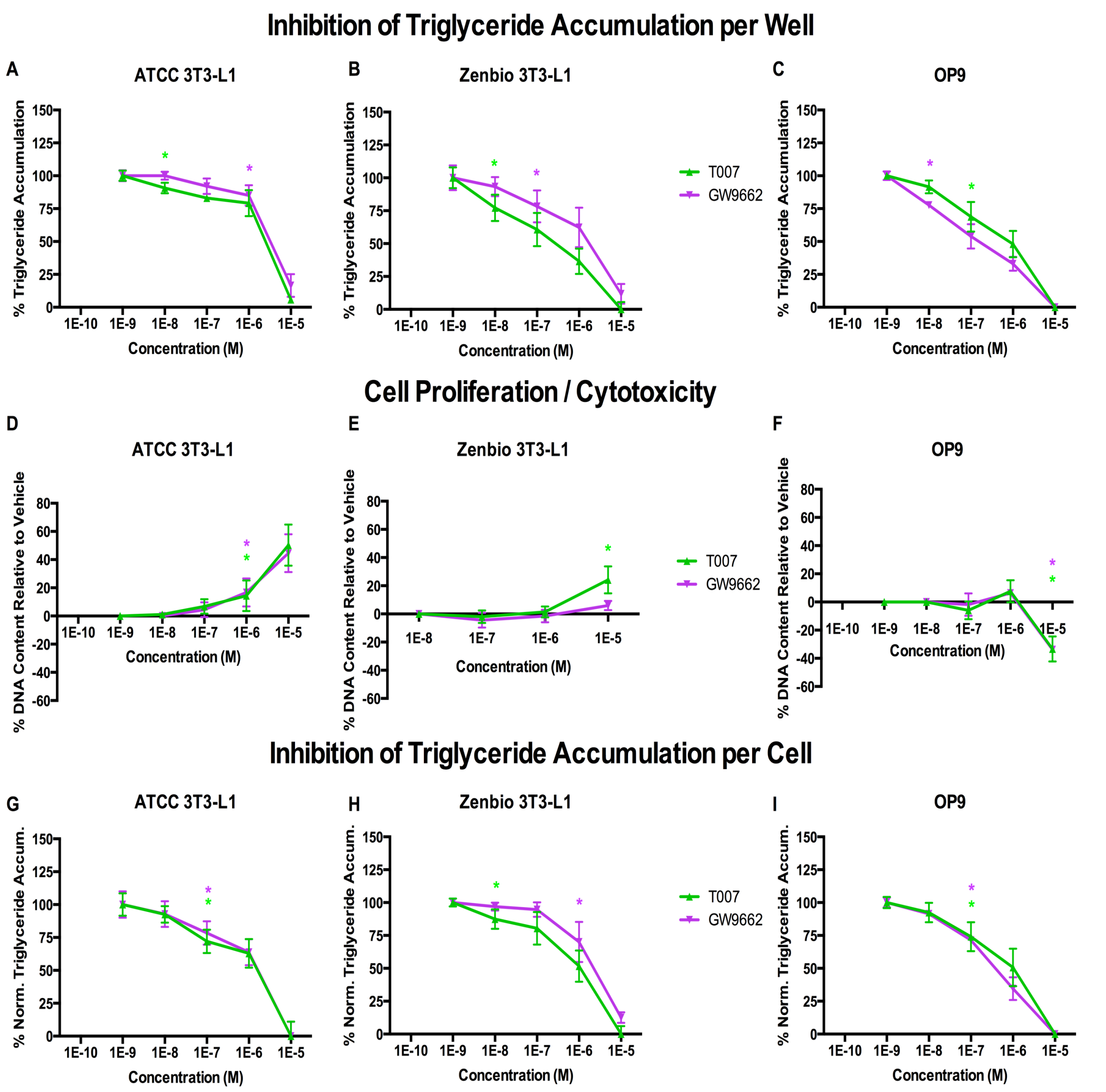

Supplemental Figure 5: **Negative Controls Induce Varied Adipogenic Activities Between Cell Lines.** *ATCC 3T3-L1, Zenbio 3T3-L1, and OP9 cells were differentiated as described in Methods and assessed for adipocyte differentiation (Nile Red staining of lipid accumulation) and cell proliferation (Hoechst staining) following seven days (OP9) or ten days (3T3-L1) of treatment with negative control chemicals. Percent raw triglyceride accumulation per well relative to half maximal rosiglitazone response for T0070907 (T007), and GW9662 in ATCC 3T3-L1 cells (A), Zenbio 3T3-L1 cells (B), and OP9 cells (C). Increase (cell proliferation) or decrease (potential cytotoxicity) in DNA content relative to half maximal rosiglitazone for test chemicals in ATCC 3T3-L1 cells (D), Zenbio 3T3-L1 cells (E), and OP9 cells (F). Percent normalized triglyceride accumulation per cell (normalized to DNA content) for test chemicals in ATCC 3T3-L1 cells (G), Zenbio 3T3-L1 cells (H), and OP9 cells (I). T007/GW9662 inhibition is of half maximal rosiglitazone in each respective cell line, set to 100% for complete absence of inhibition and deceasing to 0% triglyceride accumulation at 100% inhibition. Data presented as mean ± SE from three independent experiments. * indicates lowest concentration with significant increase in response over vehicle control, p<0.05, as per one-linear mixed effect model in SAS.*
